# Immunohistochemical Expression of p27^Kip1^, p57^Kip2^, Cyclin D1, Nestin, and Ki-67 in Ependymoma

**DOI:** 10.3390/brainsci12020282

**Published:** 2022-02-17

**Authors:** Shahad Iqneibi, Jamil Nazzal, Basma Owda, Hala Sultan, Runa Amoudi, Justin Z. Amarin, Sura Al-Ghnimat, Mamoun Ahram, Maysa Al-Hussaini

**Affiliations:** 1 School of Medicine, The University of Jordan, Amman 11942, Jordan; shahad.iqneibi@yahoo.com (S.I.); jamil.nazzal@outlook.com (J.N.); basmaowda@outlook.com (B.O.); lalasultan@hotmail.com (H.S.); runa_a@hotmail.com (R.A.); 2 Office of Scientific Affairs and Research, King Hussein Cancer Center, Amman 11941, Jordan; justinzamarin@gmail.com (J.Z.A.); sa.12608@khcc.jo (S.A.-G.); 3 Department of Physiology and Biochemistry, School of Medicine, The University of Jordan, Amman 11942, Jordan; m.ahram@ju.edu.jo; 4 Department of Pathology and Laboratory Medicine, King Hussein Cancer Center, Amman 11941, Jordan

**Keywords:** cyclin D1, cyclin-dependent kinase inhibitor p27, cyclin-dependent kinase inhibitor p57, ependymoma, Ki-67 antigen, nestin

## Abstract

p27 and p57 are tumor suppressors that are dysregulated in many cancers. We investigated the immunohistochemical expression of p27 and p57 in ependymoma, with a secondary emphasis on cyclin D1, nestin, and Ki-67. Sixty-five patients diagnosed with ependymoma were included. Clinical and tumoral data were retrieved, and the expression of p27, p57, cyclin D1, nestin, and Ki-67 was measured. Pearson’s *χ*^2^ test was used to measure associations and the Kaplan–Meier method was used for survival analysis. p27 underexpression was significantly associated with pseudopalisading necrosis in tumors with foci of necrosis (*p* = 0.004). Cyclin D1 overexpression was associated with intracranial (*p* = 0.044), recurrent (*p* = 0.022) and grade 3 tumors (*p* = 0.016); nestin overexpression was associated with supratentorial (*p* = 0.025), mitotically active (*p* < 0.001), and grade 3 tumors (*p* = 0.004); Ki-67 overexpression was associated with supratentorial (*p* = 0.044) and grade 3 tumors (*p* < 0.001) and the 3 main features of anaplasia. None of the markers were intercorrelated or predictive of overall survival. In conclusion, p27 underexpression in tumors with foci of necrosis signals a pseudopalisading pattern. Cyclin D1, nestin, and Ki-67 are useful markers in ependymoma, but evidence-based cutoff values are required to standardize this interpretation.

## 1. Introduction

Ependymomas are uncommon tumors that originate from cells of the central nervous system (CNS) along the lining of the cerebral ventricles and the central canal of the spinal cord [1]. In the United States, the age-adjusted rate of ependymoma per 100,000 population is 0.32 in children, 0.37 in adolescents and young adults, and 0.53 in older adults [2]. The most common site is the infratentorium in children and the spinal cord in adults [1]. Classification based on histopathology alone does not capture the diversity of ependymomas and relates poorly to measures of survival [3]. Indeed, ependymomas are a diverse group of entities with distinct molecular signatures that are better related to measures of survival [4].

MicroRNAs (miRNAs) are short, noncoding RNAs that regulate the expression of ~30% of human genes. These endogenous molecules are differentially expressed in human cancers and may act as oncogenes or tumor suppressor genes [5]. Indeed, molecular aberrations in ependymomas and other tumors of the CNS include overexpression or underexpression of miRNAs [6]. Previously, we showed that miR-221-3p and miR-222-3p were overexpressed in recurrent ependymomas [7]. Interestingly, Medina et al. found that miR-221 and miR-222 target the 3’ untranslated regions of p27^Kip1^ (p27, encoded by *CDKN1B*) and p57^Kip2^ (p57, encoded by *CDKN1C*) and reduce the levels of both proteins [8].

Both p27 and p57 are tumor suppressors that inhibit cyclin-dependent kinases and regulate cell cycle progression [9,10]. Consequently, p27 underexpression promotes tumorigenesis and portends worse outcomes in patients with cancer [9]. For example, p27 underexpression is associated with poorer prognosis and aggressive progression of hepatocellular carcinoma [11]. Interestingly, miR-221 controls the expression of both p27 and p57 in hepatocellular carcinoma [12]. Similar findings have been reported for tumors of the CNS. For example, a human astrocytoma cell line showed decreased cell proliferation following transfection with a recombinant adenovirus that expresses p27 (Adp27^Kip1^) [13]. In addition, p27 underexpression portends a poor prognosis in patients with astrocytoma [14,15].

Cyclin D1 (encoded by *CCND1*) is a related cell cycle protein that increases cell proliferation. Many studies have shown that tumors that overexpress cyclin D1 and underexpress p27 are more aggressive [16,17,18,19]. We previously studied the differential miRNA expression of cyclin D1–positive and cyclin D1–negative ependymomas and found that cyclin D1–positive tumors underexpressed miRNAs with tumor suppressor activity [7]. Indeed, immunohistochemical expression of cyclin D1 is associated with worse progression-free survival in ependymoma [20,21]. Other established markers of poor prognosis in ependymoma include nestin and Ki-67 [22,23]. Nestin (encoded by *NES*) is an intermediate filament that is strongly expressed by neural progenitor cells and regulated in a cell-cycle-dependent manner during neurogenesis [24]. In ependymoma, nestin expression is associated with aggressive grade 2 tumors [22]. Ki-67 (encoded by *MKI67*) is a protein that is highly expressed in proliferating cells and is widely used as a marker of proliferation in oncology [25]. A high Ki-67 proliferation index correlates with poor outcome in ependymoma [23]. Though cyclin D1, nestin, and Ki-67 are well-studied in ependymoma, data from additional cohorts are needed. In addition, the roles of p27 and p57 in ependymoma remain largely unexplored. Therefore, we investigated the immunohistochemical expression of p27 and p57 in ependymoma, with a secondary emphasis on the expression of cyclin D1, nestin, and Ki-67.

## 2. Materials and Methods

All of the patients diagnosed with ependymoma at the King Hussein Cancer Center from 2002 to 2015 were screened for eligibility. King Hussein Cancer Center, the only comprehensive cancer center in Jordan, serves almost 60% of the population [26]. All of the patients with pathologically confirmed ependymoma and sufficient tissue material for immunohistochemistry were included. Electronic medical records were accessed, and data on or related to the date of birth, sex, nationality, date of diagnosis, tumor site, metastasis at presentation, surgery, chemotherapy, radiotherapy, recurrence, survival status, date of the last follow-up, and date of death were retrieved. Pathology reports were accessed, and data on or related to mitosis, microvascular proliferation, pseudopalisading and non-palisading necrosis, clonal nodules, and grade were retrieved. Clonal nodules were defined as nodules of increased cellularity and mitosis, with or without microvascular proliferation, amid a grade 2 classical ependymoma. All of the cases were reclassified according to cIMPACT-NOW update 7 [27]. Survival data were supplemented with data from the national Civil Status and Passports Department, a branch of government whose responsibilities include curating current survival data. All of the cases were reviewed and confirmed by a neuropathologist (M. Al-Hussaini).

### 2.1. Immunohistochemistry

The BenchMark ULTRA IHC/ISH System (Ventana Medical Systems, Tucson, AZ, USA) was used to immunostain tissue samples for p27, p57, cyclin D1, nestin, and Ki-67 (Table 1). Positive and negative tissue controls were evaluated before interpreting any immunohistochemical results. For p27, p57, and cyclin D1, the percentage of tumor cells with stained nuclei was quantified. For nestin, the percentage of tumor cells with stained cytoplasm was quantified. The Ki-67 proliferation index was determined using the method of Wolfsberger et al. [23]. The percentages were enumerated within hotspots by counting at least 500 cells. Using these percentages, tumors were classified as either immunopositive or immunonegative based on a cutoff value. The cutoff value for both p27 and p57 was 10% (≥10% or <10%), and values <10% indicated immunonegativity or underexpression (mutant). For cyclin D1, the cutoff value was 50% (≥50% or <50%), and values ≥50% indicated immunopositivity or overexpression (mutant). For nestin, the cutoff value was 75% (≥75% or <75%), and values ≥75% indicated immunopositivity or overexpression (mutant). The cutoff value for the Ki-67 index was 17.5% (≥17.5% or <17.5%) based on the results of our previous study [7].

### 2.2. Statistical Methods

R (version 4.0.2) was used to perform all of the data analyses. The clinical and tumoral characteristics of all cases were described. The immunohistochemical expression of p27, p57, cyclin D1, nestin, and Ki-67 was also described. For each marker, the cases were stratified according to immunoreactivity, and Pearson’s *χ*^2^ test was used to compare the clinical and tumoral characteristics of the strata. A φ correlation matrix was generated to measure the associations between the markers. Finally, for each marker, the Kaplan–Meier method was used to plot the separate overall survival (OS) curves of patients with immunopositive and immunonegative tumors. The curves were then compared using the log-rank test, and the 5-year OS rates were estimated. Overall survival was defined as the length of time from the date of diagnosis to the date last known alive or the date of death from any cause. For all of the hypothesis tests, values of *p* ≤ 0.05 were interpreted to indicate statistical significance.

### 2.3. Ethics

The Institutional Review Board (IRB) of the King Hussein Cancer Center reviewed and approved the study protocol (19KHCC109). The IRB (King Hussein Cancer Center), which complies with the Declaration of Helsinki and the Good Clinical Practice guidelines, waived the requirement for informed consent because the study involved existing data and specimens and required no interaction with participants.

## 3. Results

### 3.1. Clinical Characteristics

In total, 65 patients were included in the final analysis; 57 (87.7%) were managed at our center and 8 (12.3%) were not. The mean age at diagnosis was 20 ± 18 years; 37 patients (56.9%) were children or adolescents (<18 years old) and 28 (43.1%) were adults (≥18 years old). Of all of the cases, 15 (23.1%) were supratentorial, 30 (46.2%) were infratentorial, and 20 (30.8%) were spinal. Moreover, 42 patients (64.6%) were male and 23 (35.4%) were female; 55 patients (84.6%) were nationals of Jordan and 10 (15.4%) were nationals of other countries in the Middle East and North Africa. The clinical characteristics of the 57 patients who were managed at our center were further studied. A total of 9 patients (15.8%) presented with metastasis. Out of the 57 patients, 26 (47.3%) underwent gross total resection, four (7.3%) underwent near-total resection, 25 (45.5%) underwent subtotal resection, and two (3.5%) were biopsied but did not otherwise undergo surgery. Overall, 14 patients (24.6%) received chemotherapy and 51 (89.5%) received radiotherapy. The disease recurred in 24 patients (42.1%).

### 3.2. Tumoral Characteristics

The histopathologic characteristics of all 65 cases were reviewed. According to cIMPACT-NOW update 7, 51 tumors (78.5%) were grade 2 and 14 (21.5%) were grade 3. The mitotic count was ≥3 (26.2%) in 17 cases; microvascular proliferation was present in 28 (43.1%) cases, and foci of necrosis were present in 41 (63.1%) cases. Foci of necrosis were non-palisading in 26 (40.0%) cases and pseudopalisading in 15 (23.1%). Clonal nodules were present in 10 (22.7%) classic ependymoma cases and absent in 34 (77.3%).

### 3.3. Immunohistochemical Analysis

**Figure 1:**
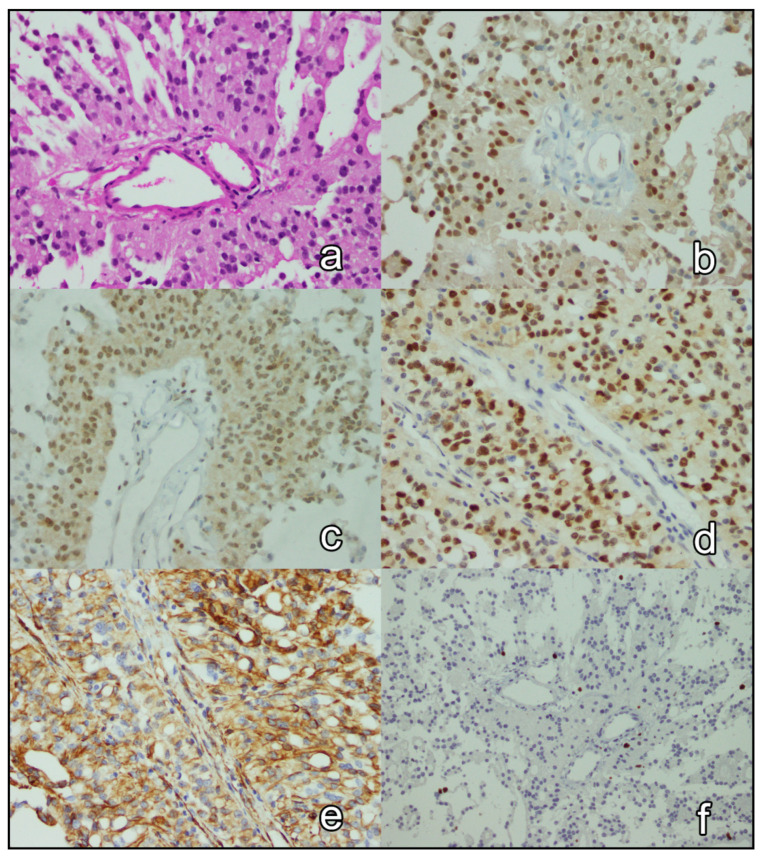
A case of grade 2 ependymoma. On H&E, cells with acidophilic cytoplasm are seen forming pseudorosettes around vessels, with no evidence of increased mitosis, microvascular proliferation, or necrosis (**a**). The nuclei of tumor cells are immunopositive for p27 (**b**), p57 (**c**), and cyclin D1 (**d**), and their cytoplasm is immunopositive for nestin (**e**). The Ki-67 proliferation index is low (**f**). Magnification X20 (**a**,**f**) and X40 (**b**–**e**).

**Figure 2:**
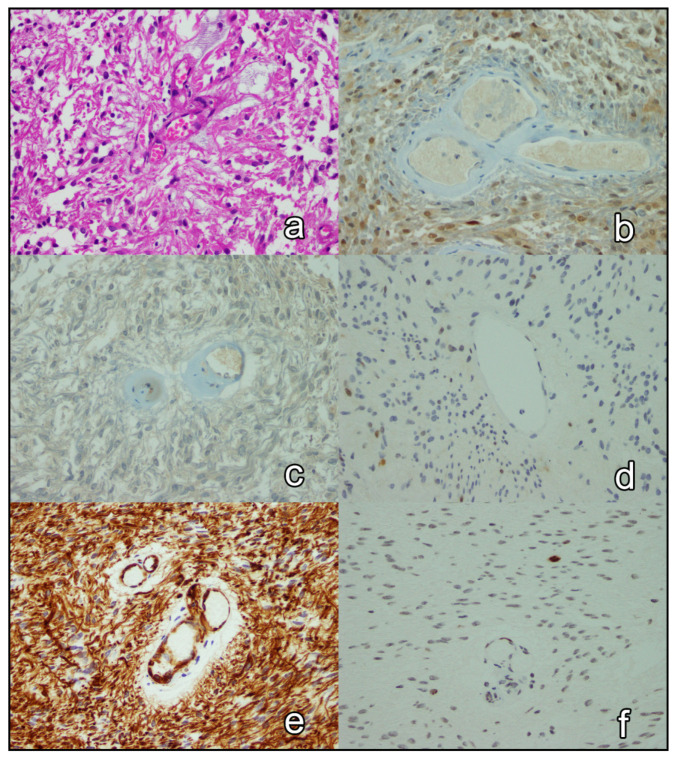
A case of grade 2 ependymoma. On H&E, a perivascular pseudorosette (**a**) is apparent. The nuclei of tumor cells are immunonegative for p27 (**b**), p57 (**c**), and cyclin D1 (**d**), and their cytoplasm is immunopositive for nestin (**e**). The Ki-67 proliferation index is low (**f**). Magnification X20 (**a**,**f**) and X40 (**b**–**e**).

**Figure 3:**
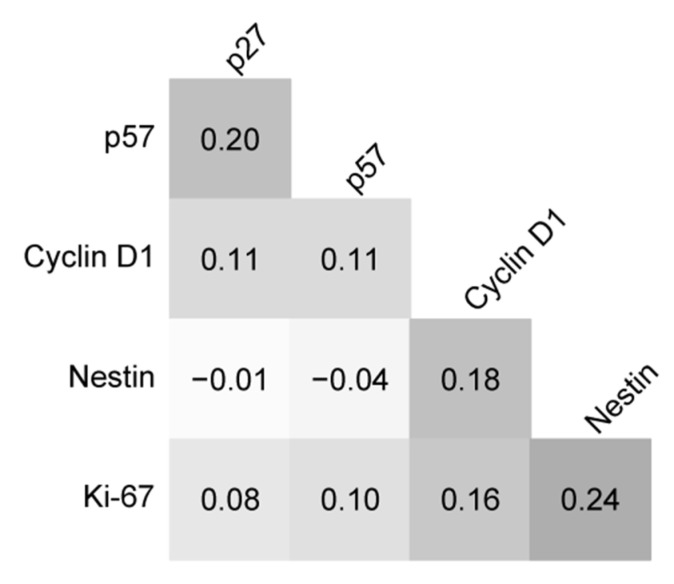
φ correlation matrix between p27, p57, cyclin D1, nestin, and Ki-67. None of the markers were statistically significantly intercorrelated.

The immunohistochemical expression of p27, p57, cyclin D1, nestin, and Ki-67 in all 65 cases was reported (Figure 1 and Figure 2 depict the results of 2 cases). The expression of p27 and p57 was lost in 16 (24.6%) cases and 42 (64.6%) cases, respectively. p27 and p57 were concomitantly lost in 13(20.0%) cases. Cyclin D1 was overexpressed in 13 (20.0%) cases. Two (3.1%) tumors overexpressed cyclin D1 and were concomitantly negative for p27 and p57. Nestin was overexpressed in 44 (67.7%) cases. Ki-67 proliferation index was ≥17.5% in 16 (26.4%) cases. Table 2 shows the associations of clinical and tumoral characteristics with the immunohistochemical expression of p27, p57, cyclin D1, nestin, and Ki-67. Briefly, p27 expression was significantly associated with the type of necrosis (*p* = 0.004) in tumors with foci of necrosis; foci pseudopalisading necrosis was seen in 77.8% (7 of 9) of p27-negative (mutant) tumors and 25.0% (8 of 32) of p27-positive tumors. Cyclin D1 expression was associated with intracranial (*p* = 0.044), recurrent (*p* = 0.022), and grade 3 tumors (*p* = 0.016); nestin expression was associated with supratentorial (*p* = 0.042), mitotically active (*p* < 0.001), and grade 3 tumors (*p* = 0.004); and, the Ki-67 proliferation index of ≥17.5% was associated with mitotically active tumors (*p* < 0.001) with microvascular proliferation (*p* = 0.003) and necrosis (*p* = 0.003), as well as supratentorial (*p* = 0.044) and grade 3 tumors (*p* < 0.001). Finally, none of the pairs of tested immunohistochemical markers were significantly associated (*p* > 0.05; Figure 3).

### 3.4. Survival Analysis

We performed survival analysis of 57 patients who were managed at our center. The patients were followed up for 360 person-years (median, 6.2 years; mean, 6.3 years). During the follow-up period, 17 (29.8%) patients died. The 5-year survival rates were 83.3% (95% CI, 64.7–100.0%) and 70.6% (95% CI, 57.3–86.9%) for patients with p27-negative and p27-positive tumors, respectively; 79.9% (95% CI, 66.6–95.8%) and 65.3% (95% CI, 47.5–89.8%) for patients with p57-negative and p57-positive tumors, respectively; 73.0% (95% CI, 60.5–88.1%) and 77.8% (95% CI, 54.9–100.0%) for patients with cyclin D1–negative and cyclin D1–positive tumors, respectively; 80.8% (95% CI, 63.4–100.0%) and 70.8% (95% CI, 57.0–87.9%) for patients with nestin-negative and nestin-positive tumors, respectively; and, 80.0% (95% CI, 67.8–94.5%) and 60.3% (95% CI, 40.0–90.9%) for patients with a low and high Ki-67 index, respectively. Survival distributions did not statistically significantly differ according to the expression of p27 (*p* = 0.92), p57 (*p* = 0.83), cyclin D1 (*p* = 0.79), nestin (*p* = 0.16), or Ki-67 (*p* = 0.53).

## 4. Discussion

We investigated the immunohistochemical expression of p27 and p57 in 65 cases of ependymoma, with a secondary emphasis on cyclin D1, nestin, and Ki-67. We found that pseudopalisading was more commonly seen in p27-negative (mutant) tumors, while non-palisading necrosis was seen in p27-positive tumors. In addition, cyclin D1–positive (mutant) tumors were more likely to be intracranial, to recur, and to be grade 3. We also found that nestin–positive tumors were more likely to be supratentorial, mitotically active, and grade 3, and tumors with a high Ki-67 index were more likely to be supratentorial and grade 3. Indeed, a high Ki-67 index was associated with all 3 features of anaplasia. Finally, we found that none of the markers were intercorrelated, and none of them predicted OS.

p27 underexpression is related to worse outcomes in some but not all cancers [9]. In this study, we found that p27 underexpression was not related to recurrence or OS. In contrast, Korshunov et al. found that p27 underexpression was related to recurrence and worse progression-free survival in high-grade but not low-grade intracranial ependymomas [28]. However, our samples are not comparable because almost one-third of our cases are spinal. In addition, the majority of their cases were grade 3, while the majority of ours were grade 2. Finally, they studied progression-free survival while we studied OS. In agreement with our results, Alexiou et al. studied 13 cases of pediatric infratentorial ependymoma and found no relation between p27 expression and OS [29]. However, their analysis may have been unable to detect a relationship because of the small sample size.

Korshunov et al. also found an association between p27 expression and grade, a finding which we did not reproduce [28]. Aligning with our results, Alexiou et al. and Schiffer et al. did not find an association between p27 expression and tumor grade [29,30]. However, again, the results must be interpreted with caution because of the small sample sizes. We found that p27-negative tumors were no more or less likely to have foci of necrosis. However, the type of necrosis was usually pseudopalisading in p27-negative tumors with foci of necrosis. Conversely, in p27-positive tumors with foci of necrosis, the type of necrosis was usually nonpalisading. This finding is clinically relevant because pseudopalisading necrosis is associated with anaplasia [31]. p27 expression was otherwise not associated with any clinical or tumoral characteristics.

In many human cancers, p57 is downregulated both transcriptionally and translationally. This downregulation portends worse outcomes in some cancers [32]. To our knowledge, p57 expression has not previously been studied in ependymoma. In this study, we measured the expression of p57 in ependymomas and found that the majority underexpressed p57. However, p57 expression was not associated with any clinical or tumoral characteristics and did not predict OS. Therefore, our results support the role of p57 as a tumor suppressor but are otherwise clinically insignificant. Interestingly, Akaishi et al. studied the expression of p57 in astrocytomas and concluded that p57 alone is insufficient to arrest cell proliferation; rather, other cell cycle proteins must be regulated in tandem to produce clinically meaningful effects [33]. Therefore, we recommend a further assessment of the diagnostic and prognostic value of a panel of cell cycle proteins with a special emphasis on interaction effects.

Cyclin D1 is a well-established oncogene that is overexpressed by many cancers [34]. We found that cyclin D1 was overexpressed in 20.0% of ependymomas; however, overexpression did not affect OS. In support, Zamecnik et al. and de Andrade et al. found that cyclin D1 overexpression predicted progression-free survival but not OS [20,21]. de Andrade et al. also showed that tumors that overexpress cyclin D1 were more likely to be intracranial, to recur, and to be grade 3 [21]. We reproduced all three findings. We also found that nestin was more likely to be overexpressed in supratentorial, mitotically active, and grade 3 tumors, which is consistent with the seminal report of Milde et al. [22]. However, they found that nestin overexpression predicts worse OS, a finding that we did not reproduce. Finally, we found that a high Ki-67 index favored grade 3 tumors and was associated with all 3 main features of anaplasia—namely high mitotic activity, microvascular proliferation, and necrosis. Though our results are generally consistent with the seminal report of Wolfsberger et al., we did not reproduce the finding that Ki-67 overexpression predicts worse OS [23].

Our study has several limitations. First, ratings of immunohistochemical expression may vary between observers. To address this limitation, two members of the study team (M. Al-Hussaini and S.A.) rated the immunohistochemical expression of the markers, and disagreements were resolved by consensus. Second, the cutoffs we selected to define immunopositivity and immunonegativity were not evidence-based; we are not aware of any evidence to substantiate the choice of any cutoff value for these markers. Establishing a cutoff value for the Ki-67 proliferation index is difficult because of the variation in staining intensity and the lack of standardized quality control measures. We recommend a study to establish a standard cutoff score for cyclin D1, nestin, and Ki-67, which appear to be useful clinical markers. Finally, we did not control for multiplicity—which increases the probability of false significant tests—because the goal of our analyses was exploratory [35]. Therefore, our results are preliminary and must be interpreted as such.

In conclusion, p27 underexpression in tumors with foci of necrosis signals a pseudopalisading pattern, a feature of anaplasia. Otherwise, the expression of p27 and p57 in ependymoma does not appear to hold diagnostic or prognostic significance. Cyclin D1, nestin, and Ki-67 are useful markers in ependymoma, but evidence-based cutoff values are required to standardize interpretation.

## Figures and Tables

**Table 1:** Details of the immunostains used.

Antibody	Clone	Retrieval	Concentration	Company
p27^Kip1^	SX53G8	CC1 standard (64 min)	Ready-to-use	Roche
p57^Kip2^	Kp10	CC1 standard (64 min)	Ready-to-use	Roche
Cyclin D1	SP4	CC1 standard (64 min)	Ready-to-use	Roche
Nestin	NE029	CC1 mild (52 min)	Ready-to-use	Quartett
Ki-67	MIB-1	CC1 mild (48 min)	1:200	Dako

**Table 2:** Associations of clinical characteristics with the immunohistochemical expression of p27, p57, cyclin D1, nestin, and Ki-67 (*N* = 65).

	p27		p57		Cyclin D1		Nestin		Ki-67	
	−, *n* (%)	+, *n* (%)	−, *n* (%)	+, *n* (%)	−, *n* (%)	+, *n* (%)	−, *n* (%)	+, *n* (%)	−, *n* (%)	+, *n* (%)
Age group										
*<18 years*	7 (43.8)	30 (61.2)	21 (50.0)	16 (69.6)	28 (53.8)	4 (69.2)	9 (42.9)	28 (63.6)	25 (51.0)	12 (75.0)
*≥18 years*	9 (56.2)	19 (38.8)	21 (50.0)	7 (30.4)	24 (46.2)	9 (30.8)	12 (57.1)	16 (36.4)	24 (49.0)	4 (25.0)
*p value*	0.22		0.13		0.32		0.11		0.093	
Anatomic site										
*Supratentorium*	3 (18.8)	12 (24.5)	11 (26.2)	4 (17.4)	10 (19.2)	5 (38.5)	1 (4.8)	14 (31.8)	8 (16.3)	7 (43.8)
*Infratentorium*	10 (62.5)	20 (40.8)	18 (42.9)	12 (52.2)	23 (44.2)	7 (53.8)	10 (47.6)	20 (45.5)	23 (46.9)	7 (43.8)
*Spine*	3 (18.8)	17 (34.7)	13 (31.0)	7 (30.4)	19 (36.5)	1 (7.7)	10 (47.6)	10 (22.7)	18 (36.7)	2 (12.5)
*p value*	0.30		0.68		0.096		0.025		0.044	
Anatomic site, collapsed										
*Intracranium*	13 (81.2)	32 (65.3)	29 (69.0)	16 (69.6)	33 (63.5)	12 (92.3)	11 (52.4)	34 (77.3)	31 (63.3)	14 (87.5)
*Spine*	3 (18.8)	17 (34.7)	13 (31.0)	7 (30.4)	19 (36.5)	1 (7.7)	10 (47.6)	10 (22.7)	18 (36.7)	2 (12.5)
*p value*	0.23		0.97		0.044		0.042		0.068	
Metastasis at presentation										
*No*	15 (100.0)	33 (78.6)	31 (86.1)	17 (81.0)	40 (87.0)	8 (72.7)	15 (83.3)	33 (83.3)	36 (87.8)	12 (75.0)
*Yes*	0 (0.0)	9 (21.4)	5 (13.9)	4 (19.0)	6 (13.0)	3 (27.3)	3 (16.7)	6 (16.7)	5 (12.2)	4 (25.0)
*p value*	0.051		0.61		0.24		0.90		0.23	
Recurrence										
*No*	10 (66.7)	23 (54.8)	21 (58.3)	12 (47.1)	30 (65.2)	3 (27.3)	11 (61.1)	22 (56.4)	24 (58.5)	9 (56.2)
*Yes*	5 (33.3)	19 (45.2)	15 (41.7)	9 (42.9)	16 (34.8)	8 (72.7)	7 (38.9)	17 (43.6)	17 (41.5)	7 (43.8)
*p value*	0.42		0.93		0.022		0.74		0.88	
Mitotic count										
*<3*	11 (68.8)	37 (75.5)	31 (73.8)	17 (26.2)	41 (78.8)	7 (53.8)	21 (100.0)	27 (61.4)	42 (85.7)	6 (37.5)
*≥3*	5 (31.2)	12 (24.5)	11 (73.9)	6 (26.1)	11 (21.2)	6 (46.2)	0 (0.0)	17 (38.6)	7 (14.3)	10 (62.5)
*p value*	0.59		0.99		0.067		<0.001		<0.001	
Microvascular proliferation										
*Absent*	8 (50.0)	29 (59.2)	22 (52.4)	15 (47.6)	32 (61.5)	5 (38.5)	15 (71.4)	22 (50.0)	33 (67.3)	4 (25.0)
*Present*	8 (50.0)	20 (40.8)	20 (65.2)	8 (34.8)	20 (38.5)	8 (61.5)	6 (28.6)	22 (50.0)	16 (32.7)	12 (75.0)
*p value*	0.52		0.32		0.13		0.10		0.003	
Necrosis										
*Absent*	7 (43.8)	17 (34.7)	19 (45.2)	5 (21.7)	20 (38.5)	4 (30.8)	11 (52.4)	13 (29.5)	23 (46.9)	1 (6.2)
*Present*	9 (56.2)	32 (65.3)	23 (54.8)	18 (78.3)	32 (61.5)	9 (69.2)	10 (47.6)	31 (70.5)	26 (53.1)	15 (93.8)
*p value*	0.51		0.06		0.61		0.074		0.003	
Type of necrosis										
*Nonpalisading*	2 (22.2)	24 (75.0)	12 (52.2)	14 (77.8)	22 (68.8)	4 (44.4)	7 (70.0)	19 (61.3)	19 (73.1)	7 (46.7)
*Pseudopalisading*	7 (77.8)	8 (25.0)	11 (47.8)	4 (22.2)	10 (31.2)	5 (55.6)	3 (30.0)	12 (38.7)	7 (26.9)	8 (53.3)
*p value*	0.004		0.09		0.18		0.62		0.091	
Nodules										
*Absent*	7 (63.6)	27 (81.8)	20 (74.1)	14 (82.4)	29 (76.3)	5 (83.3)	12 (70.6)	22 (81.5)	30 (78.9)	4 (66.7)
*Present*	4 (36.4)	6 (18.2)	7 (25.9)	3 (17.6)	9 (23.7)	1 (16.7)	5 (29.4)	5 (18.5)	8 (21.1)	2 (33.3)
*p value*	0.21		0.52		0.70		0.40		0.50	
Grade										
*II*	12 (75.0)	39 (79.6)	32 (76.2)	19 (82.6)	44 (84.6)	7 (53.8)	21 (100.0)	30 (68.2)	45 (91.8)	6 (37.5)
*III*	4 (25.0)	10 (20.4)	10 (23.8)	4 (17.4)	8 (15.4)	6 (46.2)	0 (0.0)	14 (31.8)	4 (8.2)	10 (62.5)
*p value*	0.70		0.55		0.016		0.004		<0.001	

## Data Availability

The data presented in this study are available on request from the corresponding author.
